# A Broken Trust: Lessons from the Vaccine–Autism Wars: *Researchers long ago rejected the theory that vaccines cause autism, yet many parents don't believe them. Can scientists bridge the gap between evidence and doubt?*

**DOI:** 10.1371/journal.pbio.1000114

**Published:** 2009-05-26

**Authors:** Liza Gross

**Affiliations:** Senior Science Writer/Editor, *PLoS Biology*, Public Library of Science, San Francisco, California, United States of America

## Abstract

Researchers long ago rejected the theory that vaccines cause autism, yet many parents don't believe them. Can scientists bridge the gap between evidence and doubt?

Until the summer of 2005, Sharon Kaufman had never paid much attention to the shifting theories blaming vaccines for a surge in reported cases of autism. Kaufman, a medical anthropologist at the University of California, San Francisco, knew that the leading health institutions in the United States had reviewed the body of evidence, and that they found no reason to think vaccines had anything to do with autism. But when she read that scientists and public officials who commented on the studies routinely endured malevolent emails, abusive phone calls, and even death threats, she took notice.

“Hecklers were issuing death threats to *spokes*people,” Kaufman exclaims, “people who simply related the scientists' findings.” To a researcher with a keen eye for detecting major cultural shifts, these unsettling events signaled a deeper trend. “What happens when the facts of bioscience are relayed to the public and there is disbelief, lack of trust?” Kaufman wondered. “Where does that lead us?”

Struck by how the idea of a vaccine–autism link continued to gain cultural currency even as science dismissed it, Kaufman took a 26-month hiatus from her life's work on aging and longevity to investigate the forces fueling this growing divide between scientists and citizens (see Figure 1). She wanted to understand how parents thought about risk and experts, how these attitudes shaped parents' decisions about vaccination, and what the vaccine wars might teach us about the long-term erosion of public trust in science.

**Figure 1:**
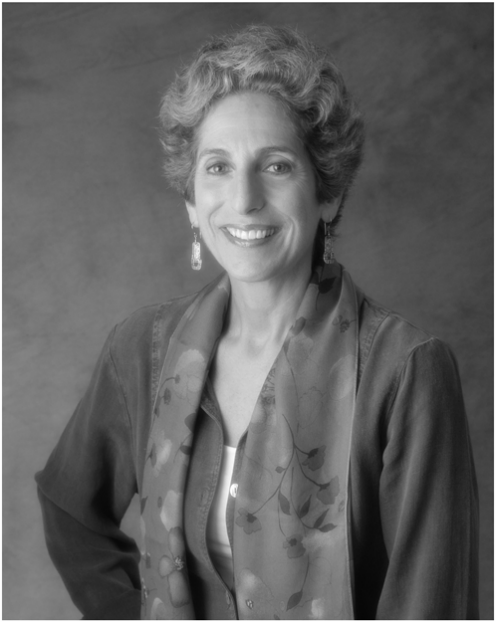
Sharon Kaufman. For most of her academic career, Kaufman, a professor of medical anthropology at the University of California, San Francisco, has studied major cultural trends related to health and aging. She saw the vaccine–autism controversy as an opportunity to understand how cultural factors shape issues of trust, risk, and responsibility as they relate to science. (Photo credit: Eliot Khuner).

Key events in the US and Britain led parents in both countries to favor different, unproven vaccine–autism theories. In the UK, confidence in the measles, mumps, rubella (MMR) vaccine plummeted after British gastroenterologist Andrew Wakefield held a press conference to promote his hypothesis that the measles virus caused a leaky gut, sending toxic substances into the bloodstream and, ultimately, the brain. Separating the MMR into three individual vaccines would be safer, he said. Wakefield's idea expanded on a finding of intestinal disease in children with autism that was published in a now discredited 1998 *Lancet* paper [1]. At press time of this Feature, Wakefield faces charges of serious professional misconduct before the General Medical Council (GMC) for allegedly violating ethical research practices on several counts. The GMC is also investigating allegations that Wakefield failed to disclose conflicts of interest—including a pending patent on a rival measles vaccine [2]. (He has denied any wrongdoing.)

In the US, fears centered around the ethylmercury-containing preservative thimerosal after a 1999 government report revealed that three childhood vaccines—diphtheria, tetanus, acellular pertussis (DTaP); *Haemophilus influenzae* type b (Hib); and hepatitis B—might expose infants to more mercury than anyone had realized. (Thimerosal, 49.6% ethylmercury by weight, was never in vaccines with live attenuated virus, including MMR.) Based on this finding, a speculative article published in a fringe medical journal spawned the theory that autism might be a form of vaccine-induced mercury poisoning.

Now, more than ten years after unfounded doubts about vaccine safety first emerged, scientists and public health officials are still struggling to set the record straight. But as climate scientists know all too well, simply relating the facts of science isn't enough. No matter that the overwhelming weight of evidence shows that climate change is real, or that vaccines don't cause autism. When scientists find themselves just one more voice in a sea of “opinions” about a complex scientific issue, misinformation takes on a life of its own.

## Evidence-Resistant Theories

Knowing that fears about MMR could easily spread in America, US public health officials had acted quickly to address festering doubts about vaccines. Officials at the Centers for Disease Control and Prevention (CDC), hoping to allay ongoing concerns that the agency couldn't objectively monitor vaccine safety while also advocating immunization, had asked the nation's leading independent advisor on science and health policy, the Institute of Medicine (IOM), for help. The IOM convened a safety review panel in 2000—explicitly excluding experts with a vested interest in vaccine safety—to address “topics of immediate and intense concern” [3].

In its first review, the IOM panel found no evidence of a causal relationship between MMR and autism “at the population level,” but couldn't rule out the possibility that it might contribute to autism spectrum disorders (ASDs) in a subset of children. Moving on to thimerosal, the panel determined that the available evidence was “inadequate to accept or reject a causal relationship between thimerosal and the disorders of autism, attention deficit, and speech and language delay” [4].

Meanwhile, CDC scientists continued their investigations of vaccine safety—prompting an angry backlash. After releasing a 2003 study of more than 140,000 children that showed no relationship between thimerosal and autism, the CDC received such disturbing threats that agency officials called in federal investigators [5]. (The CDC split its advocacy and safety monitoring branches in 2005 in an effort to restore public trust.)

By 2004, the IOM panel had reviewed over 200 epidemiological and biological studies for any link between vaccines and autism. In its eighth and final report, the panel unanimously determined that there was no evidence of a causal relationship between either MMR or thimerosal and autism, no evidence of vaccine-induced autism in “some small subset” of children, and no demonstration of potential biological mechanisms. Considering the matter resolved, the panel recommended that “available funding for autism research be channeled to the most promising areas” [4].

The report should have delivered the final blow to the vaccine–autism theories. Instead, it gave anti-vaccine activists a new target. An online group called Parents Requesting Open Vaccine Education—or PROVE, a not-so-subtle challenge to scientists to “prove” that vaccines don't cause autism—posted a roundup of parents' groups denouncing the IOM panel as “riddled with conflicts of interest” and urged parents to spread the word that panelists conspired “to sweep a generation of children under the rug and maintain current vaccine policy at any and all cost” [6].

**Figure 2:**
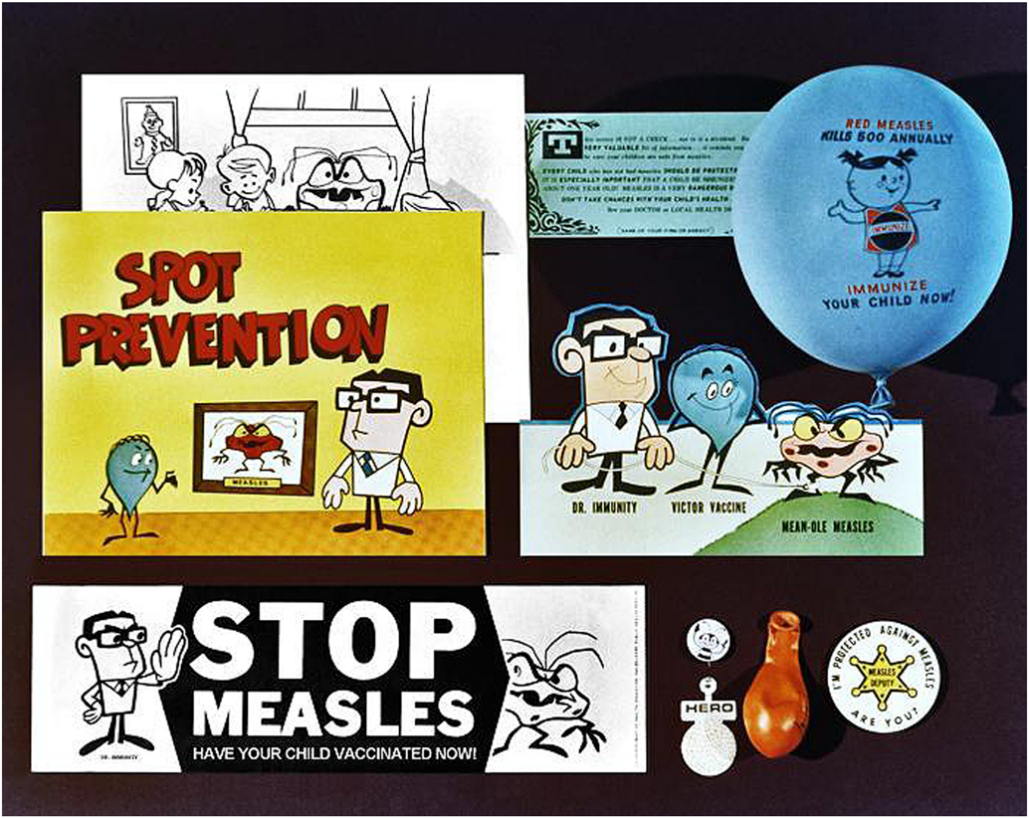
Publicizing a life-saving technology. Before 1963, when the measles vaccine became available, each year approximately 3 to 4 million cases, and an average of 450 deaths, were reported in the US. Thanks to the success of vaccination campaigns—publicized with a wealth of quirky promotional materials (above)—measles is no longer endemic in the US, though it is widespread in other countries. Last year, the US saw the largest outbreak of measles since the disease was declared eliminated in 2000; public health officials traced the majority of cases to unvaccinated Americans who imported the disease from Europe. Despite the availability of a safe, cost-effective vaccine, measles remains a leading cause of death among young children worldwide. Vaccination efforts resulted in a 74% global reduction in measles deaths between 2000 and 2007, according to the World Health Organization, yet 197,000 children died in 2007—that's nearly 540 a day.

Despite overwhelming evidence that vaccines don't cause autism, one in four Americans still think they do [7]. Not surprisingly, the first half of 2008 saw the largest US outbreak of measles—one of the first infectious diseases to reappear after vaccination rates drop—since 2000, when the native disease was declared eliminated (see Figure 2). Mumps and whooping cough (pertussis) have also made a comeback. Last year in Minnesota, five children contracted Hib, the most common cause of meningitis in young children before the vaccine was developed in 1993. Three of the children, including a 7-month-old who died, hadn't received Hib vaccines because their parents either refused or delayed vaccination.

The same trends have played out in Britain, where one in four parents told pollsters in 2002 that they believed “the weight of scientific evidence supports a link between MMR and autism” [8]. Though state law in the US requires that children be vaccinated to enter school or daycare (although parents may cite philosophical and religious reasons to claim exemptions), vaccination is not compulsory in Britain, and vaccination rates for MMR there dropped from 92% in 1998 to 80% by 2003. Although rates climbed back to 85% in 2006, England and Wales last year saw 1,000 measles cases before winter, breaking a ten-year record [9]. (Immunization rates for other childhood vaccines in Britain were largely unaffected by the MMR scare.)

Outbreaks in both countries involved primarily children who had received only one of the two recommended MMR shots or had not been vaccinated at all. US health officials traced the vast majority of 2008's measles cases to unvaccinated Americans who contracted the infection in Europe—and noted that the spike was due not to a large number of imported cases, but to increased viral transmission among unvaccinated children after importation into the US.

## Seeds of Doubt

Kaufman sees the enduring belief in the vaccine–autism theory as an example of what Ludwik Fleck, a clinical microbiologist with a passion for epistemology, called “an event in the history of thought”—a critical step in the way the perception of a scientific fact changes [10]. In the US, that first step came in the form of a simple legislative action that produced new information about what was in vaccines—and quickly fed speculative theories linking them to autism.

In 1997, a US congressman from New Jersey inserted into a funding bill a provision that gave the Food and Drug Administration (FDA) two years to measure levels of mercury in all products under its jurisdiction, and release its findings to Congress and the public. The FDA's analysis revealed that because several new vaccines were added to the immunization schedule after 1988, some infants could be exposed to as much as 187.5 micrograms of ethylmercury by the time they were 6 months old—if every dose of Hib, hepatitis B, and DTaP contained thimerosal [11].

Based on this new finding, says Kaufman, leading vaccine experts began to investigate the possibility that mercury in vaccines was putting kids at risk. While the ethylmercury levels exceeded the federal safety guidelines for methylmercury, which gains toxicity as it accumulates through the food chain, no guidelines existed for ethylmercury at the time. Its toxicity was largely unknown; however, there was evidence that very high doses of ethylmercury could cause neurological damage. It was also known that methylmercury can cause subtle neurological effects in infants born to mothers who eat large amounts of fish and whale meat. Studies have since shown that ethylmercury is eliminated much faster than methylmercury and is unlikely to accumulate. But in 1999, no one knew what dose to consider safe for the developing brain.

Given the uncertainty about ethylmercury's toxicity, Neal Halsey, director of the Institute for Vaccine Safety at Johns Hopkins University, urged vaccine policymakers at the CDC and American Academy of Pediatrics (AAP) to remove thimerosal from vaccines as a precautionary measure and to maintain public confidence in their safety. The agencies agreed, and vaccine manufacturers responded quickly; by March 2001, no children's vaccines contained thimerosal.

Anticipating the FDA's release of its findings, the AAP issued a statement explaining its decision as an effort to minimize children's exposure to mercury, asserting that “current levels of thimerosal will not hurt children, but reducing those levels will make safe vaccines even safer” [12]. Unfortunately, Kaufman says, “rather than reassuring parents, the statement fueled public fears and prompted all sorts of questions.”

To Halsey, one of the most respected figures in the vaccine world, simply ignoring the FDA's findings was not an option. He hoped the rapid response would demonstrate the government's “commitment to provide the safest vaccines possible” [13]. But it was too late for reassurances. Several months later, *Medical Hypotheses*—an unconventional journal that welcomes “even probably untrue papers”—received and later published a purely speculative article called “Autism: a novel form of mercury poisoning” [14]. Two of the authors, Sallie Bernard, a marketing consultant, and Lyn Redwood, a nurse, had just launched the parents' advocacy group SafeMinds to promote their thimerosal hypothesis. Although their now debunked theory appeared in a journal that openly eschews peer review and evidence-based observations, several parent advocacy groups still cite it as evidence that mercury in vaccines causes autism.

No one disputes that methylmercury can cause subtle neurological effects under specific conditions. But “these effects were grossly exaggerated,” Halsey says. “It was a very large leap of logic to the hypothesis that thimerosal caused autism.”

Had the discovery about thimerosal come at a different time, it might have gone unnoticed, suggests Jeffrey Baker, a pediatrician and the director of the Program in the History of Medicine at Duke University. He argues that rising autism rates, an expanded vaccine schedule, and contemporary attitudes toward environmental risk combined to create what he terms “a perfect storm” [15].

Since the 1980s, autism diagnoses in the US rose from about 0.47 per 1,000 children to about 6.7 per 1,000 today—about 1 in 150 kids. There's a perception that environmental factors explain this rapid increase, says Baker, but you don't have to go back very far to see how much the definition has expanded since Leo Kanner first described autism in 1943 (see Box 1). Asperger disorder wasn't even part of the classification scheme until 1994. “Some people say that Asperger's accounts for 50% of cases,” says Simon Baron-Cohen, director of the Autism Research Centre at Cambridge University. “If that's true, that's added at least half of the increase.”

Parents who think environmental factors are behind rising rates of autism see vaccines as the most obvious environmental exposure to have changed, Baker says. In 1983, infants were vaccinated against seven diseases; today, they receive 14 vaccinations, for a total of 26 shots by age two. “This is the single most important factor that drives parents to suspect vaccines,” Baker says.

In January, Baker appeared on an Oregon radio call-in show that featured several parents who shunned vaccination. While over 95% of Oregon parents vaccinate their children, only 70% did so last year in Ashland, a small town known for its Shakespeare festival. Nearly 60% of Ashland residents polled told the CDC, in town to hear parents' concerns, they “would expect serious consequences” from vaccines. Such low vaccination rates worry public health officials because they could signal the next epicenter of an epidemic: when vaccination rates drop below a critical percentage, called the “herd immunity threshold,” infection can swiftly spread among unprotected individuals. This threshold varies depending on the vaccine and target disease; for example, the target for measles, one of the most contagious human diseases, is 90% [16].

After hearing several parents explain why they don't vaccinate, Baker pointed out that parents who claim nonmedical exemptions seem to become so focused on their own children that they “lose the bigger picture,” not accepting responsibility for the impacts their actions may have on the health of the community. Reflecting on the radio show, Baker says, “it really hit me hard. Many of these parents who aren't vaccinating their children are just convinced that there's something in the vaccines that is poisoning their children.”

## Fanning Fears

The same month Kaufman learned that vaccine experts were getting death threats, an inflammatory piece alleging a dark conspiracy to cover up a vaccine–autism “scandal” ran simultaneously in *Rolling Stone* and the online magazine *Salon*—both of which subsequently corrected *“*several inaccuracies.” Written by Robert F. Kennedy, Jr., son of the slain US senator and presidential candidate, “Deadly Immunity” accused government officials of concealing evidence that mercury in vaccines “may have caused autism in thousands of kids” to protect drug companies from lawsuits.

The article came on the heels of a book called *Evidence of Harm,* by David Kirby, that dramatized the story of a small group of parents who “never abandoned their ambition to prove that mercury in vaccines is what pushed their children, most of them boys, into a hellish, lost world of autism.” Among the parents profiled were *Medical Hypotheses* authors Bernard and Redwood. That summer, Kennedy and Kirby hit the media circuit, leveraging RFK Jr.'s celebrity to explain why parents should fear vaccines. Remarkably, the major US public health institutions—including the Surgeon General, Department of Health and Human Services (DHHS), and National Institutes of Health—made no effort to reassure the public that vaccines are safe and could not cause the complex neurodevelopmental problems associated with autism. As Kennedy and Kirby trumpeted their largely uncontested claims, more parents filed lawsuits in federal court claiming that vaccines injured their children.

By June 2007, the parents of nearly 5,000 children with autism had sued for compensation under the Vaccine Injury Compensation Program (VICP). The program was created in 1986, after numerous lawsuits prompted by a pertussis vaccine scare forced manufacturers to flee what they considered a low-profit, high-liability market. It aimed to safeguard the nation's vaccine supply by limiting companies' liability while compensating those who experienced an adverse reaction. Although vaccines can cause several known side effects (listed in a vaccine injury table), including anaphylactic shock and even death, such events are extremely rare. For example, the risk of a serious allergic reaction, the most severe side effect for MMR, is less than 1 in a million. The risks of not vaccinating are far greater: before the measles vaccine became available in the US in the mid-1960s, 450 people died and 4,000 suffered acute inflammation of the brain each year. DHHS doctors decide whether a “table injury” was likely caused by a vaccine. Claims regarding conditions that are not listed in the table, like autism, are heard by lawyers.

The DHHS conceded in November 2007 that vaccines aggravated an underlying mitochondrial disorder in the baby girl of a Georgia couple, Terry and Jon Poling, ultimately causing “regressive encephalopathy with features of autism spectrum disorder.” Their decision was in line with previous table injury rulings that a measles-containing vaccine can exacerbate an existing encephalopathy—in this case, caused by a mitochondrial enzyme deficit. The condition shares symptoms with ASD, but is distinct.

The previous year, a case study published in the *Journal of Childhood Neurology* [17] described developmental regression and mitochondrial dysfunction in a child with autism. Jon Poling, a neurologist, was the lead author. He failed to disclose that the patient was his daughter or that he had a claim pending before the vaccine court [18]. Although the DHHS did not concede that vaccines contributed to autism, the Polings told CNN in March 2008 that the “case may well signify a landmark decision with children developing autism following vaccinations.”

Activists welcomed the case as proof that vaccines cause autism and several mainstream news outlets reported their opinions as a legitimate side of the ongoing “controversy.” In April of 2008, CNN's Larry King hosted a show on the vaccine–autism “debate” featuring Jenny McCarthy, a celebrity “autism mom” promoting a book about her son Evan's “recovery” from autism. McCarthy told King that she speaks to thousands of moms every weekend who relay the same experience: “I came home, he had a fever, he stopped speaking, and then he became autistic.” “It's time to start listening to parents who watched their children descend into autism after vaccination,” she urged, because “parents' anecdotal information is science-based information.” McCarthy said the Poling decision proved that “vaccines can trigger autism.” No scientists were on hand to challenge her.

**Figure 3:**
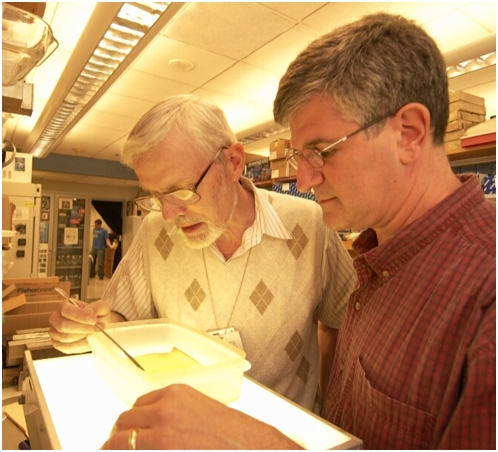
From heroes to villains. When researchers announced in 1955 that a nationwide trial showed that the first polio vaccine was safe and effective, inventor Jonas Salk was greeted as a national hero. Today, rotavirus vaccine inventor Paul Offit (right, with co-inventor H. Fred Clark) routinely endures vitriolic attacks on his credibility, along with death threats, for defending the safety of vaccines. (Photo credit: The Children's Hospital of Philadelphia).

“There's a lot of good autism research out there,” says Paul Offit, chief of infectious diseases at Children's Hospital of Philadelphia and head of the hospital's Vaccine Education Center (see Figure 3). “But you never hear about it because the anti-vaccine movement has taken this issue hostage.” Offit has turned down requests to appear on any show with McCarthy. “Every story has a hero, victim, and villain,” he explains. “McCarthy is the hero, her child is the victim—and that leaves one role for you.”

Offit's outspoken defense of vaccines, and especially his recent book, *Autism*'*s False Prophets,* has made him public enemy number one to many who think vaccines harmed their children. Even before writing the book, Offit's advocacy work earned him hate mail and death threats. His critics especially malign him for co-inventing and patenting the rotavirus vaccine, developed after a 25-year quest to prevent a disease that annually kills 600,000 children worldwide. “If you want to make a vaccine, you have to go to a pharmaceutical company,” he says. “But that instantly makes you evil.”

## A Bridge Too Far?

Kaufman sees the persistence of the vaccine–autism theory as a consequence of how individuals manage risk in modern society. People must trust experts to protect them from risk, whether they're getting on an airplane or vaccinating their kids, she explains. When faith in experts erodes, personal responsibility prevails. “People think if you blindly follow experts, you're not taking personal responsibility,” she adds.

Offit blames the media for keeping the myth alive by following the “journalistic mantra of ‘balance,’ ” perpetually presenting two sides of an issue even when only one side is supported by the science. And shows like “Larry King Live” have been “just awful on this issue,” he adds, placing ratings and controversy above public health by repeatedly giving McCarthy and other “true believers” a platform to peddle fear and misinformation. But Offit also wishes scientists would do a better job of communicating theoretical risk and the difference between coincidence and causation. Once you raise the notion of a possibility of harm, he says, “it's hard for people to get that notion out of their head.”

Kaufman thinks the problem is more immediate than bridging the gap between lay and expert understanding of risk. Parents treated theoretical risk as fact even as scientists tested, and ultimately rejected, the possibility that thimerosal might harm children. Thinking the institutions that were supposed to protect them from risk failed, Kaufman says, people now do their own research. But instead of leading to more certainty, she explains, “collecting more information actually increases doubt.”

With the explosion of “contrary” expertise online, Kaufman says, “many parents see even the most respected vaccine experts' perspective on the issue as just one more opinion.” The bulk of antivaccination Web sites present themselves as legitimate sources of scientific information, using pseudoscientific claims and emotional appeals, according to a 2002 study in *Archives of Disease in Childhood* [19]. Making matters worse, the study found, nearly all sites adopted an “us versus them” approach, casting doctors and scientists as either “willing conspirators cashing in on the vaccine ‘fraud’ or pawns of a shadowy vaccine combine.” Parents' intuitive views about vaccines were elevated above “cold, analytical science.” Accounts of children “maimed or killed by vaccines” were common—a finding that may help explain why those who advocate immunization receive death threats.

And scientists on TV and radio are hard-pressed to compete with the emotional appeals of activists. It doesn't help that science can't provide what some parents are looking for: the definitive study to prove that vaccines did not cause their child's autism. “You can never say a theory's been completely disproved, but that's just the nature of science,” observes Baron-Cohen. “So for parents, that provides something to hold on to, gives hope that the theory might one day be supported.”

As well-organized groups exploit hope and fear, parents wondering about vaccines share the fruits of their online investigations—and doubts—with moms' groups, listservs, chat rooms, and friends. Even parents who ultimately decide to vaccinate, Kaufman says, “only feel safe if they're on some sort of schedule that isn't set by science.” “Dr. Bob” Sears wrote a book that gives parents a formula to delay, withhold, separate, or space out their vaccines: *The Vaccine Book: Making the Right Decision for Your Child* sold over 100,000 copies in just two years.

These untested, “made-up” schedules just increase the window of risk for children by exposing them to potentially deadly vaccine-preventable diseases with no benefit, warns Offit. Though overall vaccination rates in the US are high, vaccine-resistant communities like Ashland have emerged in several states, including Colorado, Washington, and California, as more parents adopt alternative schedules or seek exemptions to avoid vaccination. Recent studies have shown that exempt children in Colorado were 22 times more likely to contract measles and about 6 times more likely than vaccinated children to contract pertussis, while exempt children nationwide were 35 times more likely than vaccinated children to contract measles [20].

Sadly, studies suggest that the burden of lowered immunization rates will likely fall disproportionately on poor people living in crowded conditions, hotbeds of disease transmission, and exacerbate existing health disparities among minority populations—where kids go unvaccinated not by choice but because of limited access to health services. Exemptions also pose a threat to children who can't be vaccinated because of a medical condition or who didn't mount an immune response to the vaccine, as well as to hundreds of thousands of people on chemotherapy, recovering from organ transplants, or struggling with compromised immunity.

Information technology has transformed the way trust and knowledge are produced, Kaufman says: “Scientists have to consider their role in this changed landscape and how to compete with these other sources of knowledge.” As science chips away at the genetic sources of this collection of conditions we call autism, she adds, it will chip away at the idea of a connection between vaccines and autism.

Until researchers get a better handle on the causes of autism, Baker thinks scientists need to find a way to make dry scientific results as compelling as anecdotal case studies. The studies that are “most elegant to a scientist,” he says, are just much harder for most parents to understand than what happens to an individual child.

Rachel Casiday, a medical anthropologist at the Centre for Integrated Health Care Research at Durham University, UK, who studied British parents' attitudes toward MMR, says scientists should not underestimate the importance of narrative. People relate much more to a dramatic story—“he got his vaccination, he stopped interacting, and he hasn't been the same since”—than they do to facts, risk analyses, and statistical studies. “If you discount these stories, people think you have an ulterior motive or you're not taking them seriously,” she explains. Casiday suggests providing an alternative, science-based explanation or relating emotionally compelling tales about counter-risk—such as helplessly watching a young child die of a vaccine-preventable disease—in the same narrative format.

McCarthy emerged as a hero for some parents by telling her story. Personal stories resonate most with those who see trust in experts as a risk in itself—a possibility whenever people must grapple with science-based decisions that affect them, whether they're asked to make sacrifices to help curb global warming or vaccinate their kids for public health. Researchers might consider taking a page out of the hero's handbook by embracing the power of stories—that is, adding a bit of drama—to show that even though scientists can't say just what causes autism or how to prevent it, the evidence tells us not to blame vaccines. As news of epidemics spreads along with newly unfettered infectious diseases, those clinging to doubt about vaccines may come to realize that several potentially deadly diseases are just a plane ride, or playground, away—and that vaccines really do save lives.

**Box 1. Autism at a Glance**

**Figure 4:**
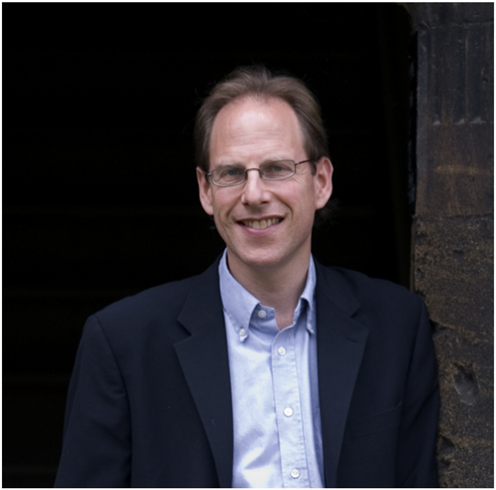
Simon Baron-Cohen. (Photo credit: Brian Harris).

Autism spectrum disorders (ASDs) are a collection of conditions characterized by stereotyped behaviors and narrow interests and pervasive problems with communication and social interactions. Symptoms typically emerge before age three and range from a severe form, called autistic disorder, to a much milder form, Asperger disorder. Though researchers can't point to any one cause of autism, mounting evidence implicates genetic factors. “We're seeing a new gene association published almost every month,” says Simon Baron-Cohen (see Figure 4)**.** “We know it's not a single-gene disorder, but we don't know if it's ten genes or a hundred genes.” Researchers also don't know how these genes function or interact with environmental factors. Preliminary evidence suggests that autism may result from disruptions in brain development caused by defects in genes involved in regulating brain growth and neuron communication [21].

In a 100-word definition published in the *British Journal of Psychiatry* last year [22], Baron-Cohen noted that children with Asperger disorder have average or above average IQ and “average or even precocious age of language onset.” In ASD, he wrote, many areas within the “social brain” are atypical, so that children may have “a profile of impaired empathy alongside strong ‘systemising’. Hence, [ASD] involves disability (when empathy is required) and talent (when strong systemising would be advantageous).” Baron-Cohen recommended developing interventions that harness systemizing to enhance empathy to help keep children on track. A number of behavioral and educational interventions may also minimize symptoms.

Because autism is far more common in males, Baron-Cohen has been exploring factors that affect sex differences in behavior to explain male vulnerability. Looking at individual variations in sociability in typically developing children, his group examined fetal testosterone (FT) levels from amniotic samples and found that the higher the children's FT levels, the less eye contact they made, the slower they developed language, and the more difficulty they had with empathy [23]. None of these differences presented at clinical levels. Now that these kids are old enough to tolerate getting into a brain scanner, Baron-Cohen can start looking at brain structure and function to see how the results relate to FT levels.

“One strategy will be to identify which brain regions differ between males and females,” he explains, “and which ones seem to be associated with testosterone. That will provide a set of regions to study in autism to see if you find the same pattern of sex differences in autism.”

Thanks to a collaboration with a group in Denmark, Baron-Cohen now has access to enough amniotic fluid samples to ask whether children diagnosed with autism have elevated FT levels. He hopes to have the results next year, but is careful to point out that FT will likely be just one piece of a very complicated jigsaw. “We're still in early days,” he says.
